# Modern Solvation Thermodynamics from Macroscopic to Microscopic Approach

**DOI:** 10.3390/e28101050

**Published:** 2026-09-24

**Authors:** Arieh Ben-Naim

**Affiliations:** Department of Physical Chemistry, The Hebrew University of Jerusalem, Edmond Safra Campus, Givat Ram, Jerusalem 91904, Israel; arieh.ben-naim@mail.huji.ac.il

**Keywords:** solvation, solvation thermodynamics, solvation entropy, solvation Gibbs energy, solvation of inert solute, self-solvation, solvation of proteins

## Abstract

The concept of solvation was originally studied within thermodynamics. This may be referred to as the *macroscopic* approach to solvation. It involved the transfer of solute from the gaseous phase into the solvent under well-specified thermodynamic conditions. In 1978, a new definition of the *solvation process* was introduced. This new definition may be referred to as the *molecular* or the *microscopic* approach to the study of solvation. Once the new definition was introduced and shown to be advantageous, all the traditional definitions were rendered obsolete. With this new definition, a host of difficult problems in chemistry and biochemistry could be studied, including the mystery associated with the entropy of solvation of inert gases in water, the problems associated with protein folding, and protein self-assembly.

## 1. Introduction

The study of *solvation* has quite a lengthy history. Its history is almost identical with the history of physical chemistry. The term *solvation* is a generalization of the term *hydration*, which was used for the specific case when the solvent was water. The earliest “definition” of the concept of solvation is probably due to Gurney [1], who wrote in his book, *Ionic Processes in Solutions*, the following:

“*The interaction that takes place when an ion is introduced into a solvent is called the solvation of the ion.”*

Of course, the concept of “solvation,” as used today, does not apply only to ionic solutes, but to any solute in any solvent. Indeed, the concept of solvation involves the interaction of the solute with the solvent, but it includes all the effects that result from these interactions. Finally, the process of *introducing* the ion (or any other solute) into the solvent was not specified in Gurney’s definition. This led to multiple measures of solvation quantities based on different *processes* of *solvation*.

In the early history of physical chemistry, the process of solvation was studied exclusively within thermodynamics. This may be referred to as the *macroscopic* approach. It involved the transfer of solute from the gaseous phase into the solvent under well-specified thermodynamic conditions. We shall describe three of those “conditions” in Section 2. Within the macroscopic approach, it was not possible (in fact, it is impossible) to tell which of the various measures was the best one to convey the “interaction” between the solute and the solvent. All this changed when a new definition of the *solvation process* was introduced in 1978. This new definition is now referred to as the molecular or the microscopic approach to the study of solvation. This approach is discussed in Section 3. Once the new definition was introduced and shown to be advantageous, all the traditional definitions became obsolete. We shall describe the advantages of the microscopic approach in Section 4 and demonstrate why this approach became the only valid definition in studying solvation phenomena. In Section 5, we present a few examples of the application of solvation to specific problems, problems which could not have been dealt with within the old macroscopic approach to solvation thermodynamics.

## 2. The Traditional or the Macroscopic Approach

Traditionally, solvation phenomena were studied within thermodynamics of solution. Since most of the experimental work was performed on systems at room temperature (fixed temperature *T*) and atmospheric pressure (fixed pressure *P*), the natural thermodynamic quantity used to study such systems was the Gibbs energy (previously referred to as “*free energy*”). The process of solvation was described in terms of transferring one mole (or one molecule) from the gaseous phase (normally being an ideal gas mixture) into the liquid phase. The two phases were supposed to be at the same temperature *T* and total pressure *P*. The Gibbs energy change for such a process is written as follows:

(1) $$\Delta G\left(\begin{array}{c} g\to l\\ c_{s}^{g},\;c_{s}^{l} \end{array}\right)$$

where $c_{s}^{g}$ and $c_{s}^{l}$ are the concentrations of the solute *s* in the two phases, gaseous (*g*), and liquid (*l*), respectively. To “*standardize*” the process of solvation, people chose to transfer the solute from *g* to *l* in which the solute concentrations are equal, i.e., $c_{s}^{g}=c_{s}^{l}$. Therefore, the *standard* Gibbs energy change for this transfer process is written as follows:

(2) $${\Delta \mu }_{s}^{o,c}\left(\begin{array}{c} g\to l\\ c_{s}^{g}=\;c_{s}^{l} \end{array}\right)$$

where the Gibbs energy change ΔG was replaced by the difference in the *chemical potential* of the solute in the two phases. The superscript “*o,c*” was used to indicate the choice of a *specific standard* process of transferring the solute between the two phases, using a specific concentration scale.

The chemical potential was introduced by Gibbs and is defined as the “derivative” of the Gibbs energy with respect to the number of particles of the species *s*, at constant temperature, pressure, and the numbers of molecules of all the other species present in the system, i.e.,

(3) $$\mu_{s}={\left[\frac{\partial G\left(T,P,N_{1},\ldots ,N_{c}\right)}{\partial N_{s}}\right]}_{T,P,\;\;\;and\;all\;N_{i},\;\;\;i\neq s}$$

Note that in the very definition of the chemical potential, it is assumed that the variable $N_{s}$ is a continuous one. For details on the justification of this assumption, see Ben-Naim [2].

So far, the standard Gibbs energy of solvation does not specify the choice of the “concentration” *scale* used for $c_{s}^{g}$ and $c_{s}^{l}$. It is at this point that different scientists had different preferences for the concentration scales. The three most common concentration scales were as follows:

1. The *molar* concentration is defined as follows:

(4) $$\rho_{s}=\frac{N_{s}}{V}$$

where $N_{s}$ is the number of solute particles, and *V* is the volume of the system.

2. The mole-fraction concentration is defined as follows:

(5) $$x_{s}=\frac{N_{s}}{\sum_{i=1}^{c}N_{i}}=\frac{\rho_{s}}{\sum_{i=1}^{c}\rho_{i}}$$

3. The molar concentration, denoted by $m_{s}$, is the number of moles of the solute *s* in one kilogram of the solvent.

In this article, we shall discuss only the first two concentration scales, which were most commonly used in the study of solvation. The molality and the related “aqua-molality” were rarely used.

Once the concentration scale is chosen, one can define the so-called *standard chemical* potential corresponding to each concentration scale.

As we noted above, the solvation thermodynamics was studied only for very dilute solutions of the solute *s* in the solvent *l* in the macroscopic approach to solvation. For such solutions, it was known that the chemical potential of the solute *s* has the following general form:

(6) $$\mu_{s}=\mu_{s}^{o,c_{s}}+RT\ln c_{s}$$

Thus, the standard Gibbs energy of solvation could be defined for each of the concentration scales. We shall refer to these as the ρ*-process*, the *x*-*process*, and the *m*-*process*. These three quantities are defined for the process of transferring one mole of solute from the gaseous to the liquid phase, as in notation (2), but for the chosen concentration scales $c_{s}=\rho_{s}$, $x_{s}$, or $m_{s}$. Thus, the standard Gibbs energy of solvation for the ρ-process is defined by the following:

(7) $$\begin{array}{l} {\Delta \mu }_{s}^{o,\rho }\left(\begin{array}{c} g\to l\\ \rho_{s}^{g}=\;\rho_{s}^{l} \end{array}\right)=\mu_{s}^{o,\rho ,l}\;-\;\mu_{s}^{o,\rho ,g}\\ =\mu_{s}^{l}\left(T,P,\rho_{s}^{l}\right)\;-\;\mu_{s}^{g}\left(T,P,\rho_{s}^{g}\right)\\ =\mu_{s}^{o,\rho ,l}\;-\;\mu_{s}^{o,\rho ,g}+{\left[RT\ln\frac{\rho_{s}^{l}}{\rho_{s}^{g}}\right]}_{\rho_{s}^{l}=\rho_{s}^{g}} \end{array}$$

i.e., transferring of one mole of solute *s* from *g* to *l*, at constant *T* and *P*, and $\rho_{s}^{g}=\;\rho_{s}^{l}$.

For the *x*-process and the *m*-process, the corresponding standard Gibbs energy of solvation is defined similarly to Equation (7) except for the condition of equal mole fractions and equal molality, respectively.

In this article, we shall discuss only the first two concentration scales, which were most commonly used in the study of solvation. The molality and the related “aqua-molality” were rarely used. We shall not discuss in this article any details regarding the relationship between the various standard Gibbs energies of solvation based on the different scales. The details may be found in Ben-Naim [3]. We emphasize here that all the conventional standard quantities were defined for very dilute solutions of the solute *s* in the solvent *l*. More importantly, no one really knew what these standard quantities actually measure. In fact, it was demonstrated in Ben-Naim [3] that the standard Gibbs energy of solvation based on the mole fraction diverges to minus infinity for a solvent with a density that tends to zero. This means that ${\Delta \mu }_{s}^{o,x}\left(x-process\right)$ *cannot* be used as an adequate measure of the “interaction” between the solute and the solvent (Gurney [1]).

This situation led to the “revolution” in the field of solvation thermodynamics in 1978. It started with a new *definition* of the *solvation process*, for which all the thermodynamic quantities could be defined on a molecular level. This new, or microscopic approach, will be discussed in the next Section.

## 3. The Microscopic Approach to Solvation

The microscopic approach to solvation starts with the *general expression* for the chemical potential for any species *i* in any system:

(8) $$\mu_{i}=\mu_{i}^{*}+kT\ln\rho_{i}\Lambda_{i}^{3}$$

This is similar to the expression for the chemical potential for extremely dilute solution of a solute *s* in a solvent *l*, which we quoted in Equation (6). Here, the expression is much more general, and is valid for any concentration of the species *i*. Unlike the standard chemical potential, which is independent of the concentration *s*, the quantity $\mu_{i}^{*}$ referred to as the pseudo-chemical potential may depend on all the concentrations of all the species in the system. The quantity $\Lambda_{i}^{3}$ is referred to as the momentum partition function, or the thermal de Broglie wavelength. It will not be involved in the definition of the solvation quantities.

The term *pseudo*-chemical potential was given to $\mu_{s}^{*}$ to emphasize that this quantity is not the chemical potential of *s* in any specific (standard) state. The significance of the general split of the chemical potential into two terms is that it corresponds to introducing the solute *s* (or any species *i*) into the solution in two steps:

First, introduce the particle (which can be any atom or a molecule) *s* at a specific location $R_{s}$, and then release the particle into the solvent *l*, Figure 1.

We now define the *process* of *solvation* as the process of transferring a single particle of any species α, not necessarily a solute in a dilute solution, from a fixed position in an ideal gas phase (or in vacuum) to a fixed position in a solvent (*l*); see Figure 2. The solvent may be characterized either by the variables $T,V,N_{1},\ldots ,N_{c}$ or by the variables $T,P,N_{1},\ldots ,N_{c}$.

The pseudo-chemical potential of a molecule of species α in an ideal gas is simply:

(9) $$\mu_{\alpha }^{*ig}=kT\ln q_{\alpha }^{\;\;-\;\;1}$$

It is assumed that the internal partition function $q_{\alpha }$ is independent of the thermodynamic variables (either T,V,N or T,P,N).

The pseudo-chemical potential of the same molecule in the liquid phase is as follows:

(10) $$\mu_{\alpha }^{*l}=kT\ln q_{\alpha }^{\;\;-\;\;1}\;\;-\;\;kT\ln\langle \exp\left(\;\;-\;\;{\beta B}_{\alpha }\right)\rangle$$

Here, $\beta \;=\frac{1}{kT}\;and\;B_{\alpha }$ is the total *binding energy* of the molecule of species α to the rest of the system. Note that the average, in the last term on the right-hand side of Equation (10), is taken either in the T,V,N or T,P,N ensemble.

Since we usually assume that the internal partition function $q_{\alpha }$ is unchanged in the process of solvation, the corresponding “free energy” change (either the Helmholtz or the Gibbs energy) is given by the following:

(11) $$\;{\Delta \mu }_{\alpha }^{*}=\mu_{\alpha }^{*l}\;\;-\;\;\mu_{\alpha }^{*ig}=\;\;-\;\;kT\ln\langle \exp\left[\;\;-\;\;{\beta B}_{\alpha }\right]\rangle$$

For a dilute solution of α in the solvent, the quantity ${\Delta \mu }_{\alpha }^{*}$ becomes identical to the standard “free energy” of solvation for the ρ-process. See Ben-Naim (1987) [3] for details.

(12) $${\Delta \mu }_{\alpha }^{*}\to {\Delta G}_{\alpha }^{0}\left(\rho -process\right)$$

Note, however, that although numerically ${\Delta \mu }_{\alpha }^{*}$ becomes identical with ${\Delta G}_{\alpha }^{0}\left(\rho -process\right),\;in\;the\;limit\;of\;very\;dilute\;solutions,$ these two quantities pertain to two *different* processes. This brought about the confusion between the solvation free energy (Helmholtz or Gibbs energy) ${\Delta \mu }_{\alpha }^{*}$ and the *standard* solvation free energy.

## 4. Advantages of the New Process of Solvation

In this section, we will briefly discuss the advantages of the new measure of solvation thermodynamics over the conventional ones. This was discussed in great detail in Ben-Naim [3].

The main advantage of ${\Delta \mu }_{\alpha }^{*},\;$which was also the motivation for seeking a new process of solvation, is that it is a genuine measure of the effect of the solvent on the thermodynamic behavior of a single molecule. In the conventional approach, it was impossible to decide, within thermodynamics, which one is the best to use.

The second important advantage is that ${\Delta \mu }_{\alpha }^{*}$ may be applied to any molecule in any solvent at any concentration of the solute α. The conventional solvation quantities were defined only for dilute solutions of α in the solvent. Within the new definition of the solvation process, there is no need to make a distinction between a solute and a solvent. In fact, the new quantity ${\Delta \mu }_{\alpha }^{*}$ may be applied even to a pure liquid. This led to the new concept of *“self-solvation*,” i.e., the solvation of a molecule in its own liquid. Such a study could never be achieved with the standard approach to solvation.

Finally, we note here that all the thermodynamic quantities associated with the *solvation process* could be derived from the Gibbs or Helmholtz energy of solvation, by the standard relationships. For example, the entropy, enthalpy, and the volume of solvation can be obtained from the standard thermodynamic relationships:

(13) $$\;{\Delta S}_{\alpha }^{*}=-{\left(\frac{{\partial \Delta \mu }_{\alpha }^{*}}{\partial T}\right)}_{P},{\Delta H}_{\alpha }^{*}={\Delta \mu }_{\alpha }^{*}+T{\Delta S}_{\alpha }^{*},\;{\Delta V}_{\alpha }^{*}=-{\left(\frac{{\partial \Delta \mu }_{\alpha }^{*}}{\partial P}\right)}_{T}$$

Thus, ${\Delta S}_{\alpha }^{*}$, ${\Delta H}_{\alpha }^{*}$, and ${\Delta V}_{\alpha }^{*}$ are the entropy, enthalpy, and the volume changes for the *same process* of solvation defined in this Section. Such simple relationships for the entropy, enthalpy, and volume could not be obtained for the solvation quantities based on “standard states.” Examples are discussed in detail in Ben-Naim [3].

## 5. Some Specific Applications of the Molecular Definition of the Solvation Process

In this section, we survey a few specific problems that could not have been studied within the old macroscopic approach but that were successfully treated and solved within the new molecular approach to solvation. These are the following: (1) Solvation of water in water: a study led to a molecular definition of the structure of water and a better understanding of the anomalous properties of liquid water. (2) A study of hydrophobic interaction led to an experimental measure of the hydrophobic interaction and later revolutionized the whole field of the so-called hydrophobic effect. (3) Solvation of inert gases: the mystery of the large and negative entropy of solvation of inert solutes in water. (4) Solvation and protein: this led to the understanding of a few mysteries in protein chemistry, such as the large solubility of globular proteins in water, the protein folding problem, and the self-assembly of proteins in aqueous solutions. To the best of our knowledge, these are all the problems that were studied and that reached a reasonable solution within the microscopic theory of solvation. More details can be found in the work of Ben-Naim and Sordo [4].

### 5.1. Solvation of Water in Water and the Molecular Definition of the Structure of Water

As we have pointed out in Section 4, within the old approach to solvation thermodynamics, only extremely dilute solutions could be studied. However, within the new definition of the solvation process, there are no restrictions on the concentration of the solute. This has opened the way for the study of “self-solvation,” i.e., the solvation of a molecule in its own liquid. Such a study could never be carried out within the standard approach to solvation. The most important liquid is, of course, liquid water. The study of the self-solvation of water in pure liquid water led to a new definition of the structure of water (SOW), as well as improved our understanding of the outstanding properties of liquid water (Ben-Naim [5]). Preceding the definition of the SOW, it was necessary to define the specific pair potential for a pair of water molecules. This was achieved in the mid-1970s (Ben-Naim [6]).

Let us denote by $X_{i}$ the configuration (i.e., location and orientation) of the *i*th water molecule. The pair potential between two water molecules at a configuration $\left(X_{1},\;X_{2}\right)$ is supposed to have at least two components:

(14) $$U\left(X_{1},\;X_{2}\right)=U_{LJ}\left(R_{12}\right)+\varepsilon_{HB}G\left(X_{1},\;X_{2}\right)$$

where $U_{LJ}$ is a Lennard–Jones pair potential which is conveniently taken as the pair potential for two neon atoms. The second term on the right-hand side of Equation (14) is referred to as the hydrogen-bond (HB) part of the pair potential. It has two factors: $\varepsilon_{HB}$, which measures the strength of the HB between two water molecules, and $G\left(X_{1},\;X_{2}\right),$ which is referred to as a *geometrical function*. This function is defined as follows:

(15) $$G\left(X_{1},\;X_{2}\right)=\left\{\begin{array}{cc} 1 & whenever\;1\;and\;2\;are\;hydrogen\;bonded\\ 0 & otherwise \end{array}\right.$$

Without going into any details about its definition, it is clear that the geometrical function can be used to define the number of HBs in which the *i*th water molecule is engaged, giving a configuration $X^{N}$ of all the molecules in the system, i.e.,

(16) $$\psi_{i}\left(X^{N}\right)=\sum_{j\neq i}^{N}G\left(X_{i},\;X_{j}\right)$$

Clearly, if we use the step function in Equation (15), then $\psi_{i}\left(X^{N}\right)$ simply *counts* the number of water molecules which are hydrogen bonded to the *i*th molecule at the configuration $X^{N}$ of the system. The average of the function $\psi_{i}\left(X^{N}\right)$ over all the configurations in either the $\left(T,V,N\right)$ or $\left(T,P,N\right)$ ensemble will depend on the index *i* and is defined by the following:

(17) $$\langle \psi \rangle =\int dX^{N}\mathrm{Pr}\left(X^{N}\right)\psi_{1}\left(X^{N}\right)$$

It is easy to show that the quantity $\langle \psi \rangle$ is related to the *average number* of HBs in the system, i.e.,

(18) $$\begin{array}{l} \langle N_{HB}\rangle =\frac{1}{2}\int dX^{N}\mathrm{Pr}\left(X^{N}\right)\sum_{i=1}^{N}\sum_{j\neq i}^{N}G\left(X_{i},\;X_{j}\right)\\ =\frac{1}{2}N\int dX^{N}\mathrm{Pr}\left(X^{N}\right)\psi_{1}\left(X^{N}\right)\\ =\frac{1}{2}N\langle \psi \rangle \end{array}$$

The factor $\frac{1}{2}$ enters because in carrying out the summing over *i* and *j* we count each pair twice. Clearly, either $\langle \psi \rangle$ or $\langle N_{HB}\rangle$ may be used to define the SOW.

We now briefly mention an approximate measure of the SOW based on the knowledge of the Gibbs energy of solvation of water in water. For details, see Ben-Naim [5,6].

We start with the definition of the self-solvation Gibbs energy of water:

(19) $$\;\;{\Delta G}_{w}^{*}=\;-\;kT\ln{\langle \exp\left[\;-\;\beta B_{o}\right]\rangle }_{T,P,N}$$

Here, the average is taken in the T,P,N ensemble. The SOW is now approximately defined in terms of the difference in the self-solvation Gibbs energies of water and of heavy water:

(20) $$\;{\langle \psi_{H_{2}O}\rangle }_{0}=2\frac{{\Delta G}_{D_{2}O}^{*}\;-\;{\Delta G}_{H_{2}O}^{*}}{\varepsilon_{D}\;-\;\varepsilon_{H}}\;$$

where $\varepsilon_{D}\;and\;\varepsilon_{H}$ are the energy parameters$\;\varepsilon_{HB}$ for $D_{2}O$ and $H_{2}O,$ respectively.

Thus, we obtained a measure of the structure of water based on experimentally available solvation quantities.

### 5.2. Study of Hydrophobic Interaction Leading to an Experimental Measure of the Hydrophobic Interaction

Consider the process $\left(\infty \to R_{12}\right)$ of bringing two simple solute molecules from infinite distance to some final distance $R_{12}$, as shown in Figure 3.

In this figure, we carry out the same process in an ideal gas phase and in water. The difference in the work of the same process in the two phases is as follows:

(21) $$\begin{array}{l} {\Delta G}^{l}\left(\infty \to R\right)\;-\;{\Delta G}^{ig}\left(\infty \to R\right)=\delta G\left(\infty \to R\right)\\ ={\Delta \mu }^{*l}\left(R\right)\;-\;{\Delta \mu }^{*l}\left(\infty \right) \end{array}$$

This is easy to prove by merely looking at Figure 3. The Gibbs energy change for the entire cyclic process must be zero. Hence, we must have the following equality:

(22) $${\Delta G}^{l}\left(\infty \to R\right)-{\Delta \mu }^{*l}\left(R\right)-{\Delta G}^{ig}\left(\infty \to R\right)+{\Delta \mu }^{*l}\left(\infty \right)=0$$

Using the statistical mechanical expression for the solvation Gibbs energies on the right-hand side of Equation (21), we obtain the following:

(23) $$\begin{array}{l} \delta G\left(\infty \to R\right)={\Delta \mu }^{*l}\left(R\right)-{\Delta \mu }^{*l}\left(\infty \right)\\ =kT\ln\langle \exp\left[-\beta B_{12}\left(\infty \right)\right]\rangle -kT\ln\langle \exp\left[-\beta B_{12}\left(R\right)\right]\rangle \\ =-{\Delta \mu }_{1}^{*l}-{\Delta \mu }_{2}^{*l}+{\Delta \mu }^{*l}\left(R\right) \end{array}$$

Next, we note that ${\Delta \mu }^{*l}\left(R\right)$ does not contain the *direct* interaction energy between the two solute particles. This fact was exploited to construct a measure of the strength of the hydrophobic interaction between two real solute molecules.

In order to do this, we consider two methane molecules at infinite separation. We bring these two molecules to any distance R=1.53 Å. This process led to the following measure of the hydrophobic interactions. We first bring the two methane molecules to the distance R=1.53 Å. The solvation Gibbs energy of the pair of these molecules is denoted by ${\Delta \mu }^{*l}\left(R=1.53\;Å\right)$. Next, we replace the two methane molecules with one *real* ethane molecule. From the point of view of the solvent, the binding energy of the two methane molecules at R=1.53 Å is approximately the same as the binding energy of one real ethane molecule; hence, we write the following:

(24) $$\;{\Delta \mu }^{*l}\left(R=1.53\;Å\right)\approx {\Delta \mu }^{*l}\left(ethane\right)$$

Using this approximation, we can obtain the required measure of the strength of the hydrophobic interactions between two methane molecules at the distance of R=1.53 Å; i.e., we write the following:

(25) $$\delta G\left(\infty \to R=1.53\;Å\right)≅{\Delta \mu }^{*}\left(ethane\right)-{\Delta \mu }^{*}\left(methane\right)$$

This measure, and numerous generalizations of it, was very useful in the study of the hydrophobic interaction and later led to a revolution in the understanding of protein folding; see Section 5.4 below.

### 5.3. Solvation Thermodynamics of Inert Gas Solutes in Water

The anomalous values of the thermodynamic quantities of solvation of inert molecules or atoms were known long before the solvation process, and the corresponding thermodynamics were well defined. In the 1930s and the 1940s, it was well known that argon or methane dissolve quite sparingly in water. It was also known that the temperature dependence of the solubility of argon in water was larger than the corresponding curves for argon in some other non-aqueous solvents; see Ben-Naim [5,6].

Translating these facts into solvation thermodynamic quantities, the solubility turns into solvation Gibbs energy, and the temperature dependence of the solubility turns into the entropy and the enthalpy of solvation. In present-day terminology, we can say that the Gibbs (or Helmholtz) energy of solvation of, say, argon in water is large and positive, and that both the entropy and the enthalpy of solvation of argon in water are large and negative compared to the corresponding values for the solvation process in the typical organic solvent. The latter facts were considered a great mystery. The first attempt to interpret these findings was completed by Eley [7,8]. Eley’s interpretation was based on a lattice model for liquid water, which today is quite obsolete.

Remember that during most of the last century entropy was considered as a measure of disorder in a system (this interpretation still appears in today’s popular science books and was proven to be baseless). Negative solvation entropy meant, for many, an ordering effect of the inert atom or molecule on water.

Frank and Evans [9,10] attempted to interpret this phenomenon in terms of “increasing the structure of water” (also known as the *iceberg formation theory*). *Negative solvation entropy* meant that an inert solute, such as argon or methane, increases the *order of water*. This mystery became even greater when the entropy of solvation of, say, argon in water was found to be even *more negative* than the entropy of solvation of electrolytes such as KCl in water. One could easily argue that the strong electric field around the charged ions orders the water molecules around them. But how can an inert atom such as argon do this ordering?

Once the new definition was introduced and shown to be advantageous, it led to a modern view of the entropy of solvation of inert gases in water. In fact, the new definition of solvation together with the Kirkwood–Buff theory of solutions led to the answer to the following question: “Why would an inert solute such as argon increase the structure of water?” In other words, the mystery of the entropy of solvation of inert solutes in water was resolved. For more details on this, the reader is referred to Ben-Naim and Sordo [4]. Other problems in which the new definition of the solvation process was found useful are the problems associated with proteins, such as the solubility of proteins in water, the problem of protein folding, and protein–protein association.

### 5.4. Solvation of Protein

The solvation of proteins cannot be studied experimentally. However, one can study the solvation of proteins by theoretical methods. This has led to the understanding of a few mysteries in protein chemistry, such as the large solubility of globular proteins in water, the protein folding problem, and the self-assembly of proteins in aqueous solutions.

We will start with a well-known experimental fact: the high solubility of globular proteins, which for quite a long time was not considered a problem at all. For quite a long time, the experimental fact about the large solubility of globular proteins in water was “well understood.” It was known that a large number of hydrophilic groups are located on the surface of the protein. Because of the strong interactions between these hydrophilic groups and water, it was deduced that the solvation Gibbs energy of the protein must be large and negative, which is equivalent to a large solubility.

However, in 1949, Hughes et al. [11] published an article in which they showed that some proteins known to have a large number of hydrophilic groups were less soluble in water. In fact, they showed that in some cases, making the surface of the protein more hydrophilic causes a *reduction* in solubility. This fact was a mystery for quite some time and was only resolved in the late 1990s upon the discovery of the role of the hydrophilic interactions in the solvation of globular proteins. In a study by Wang and Ben-Naim [12], it was concluded that indeed the high solubility of globular proteins in water is due to the large number of hydrophilic groups on their surface. However, what is important is not the number of hydrophilic groups, but their specific *distance distribution* on the surface of proteins. As a simple example, demonstrating the distance dependence of the hydrophilic groups on the solubility, consider the two isomers: maleic acid and fumaric acid. Their solubilities in water are as follows: maleic acid: 78 gr/100 mL, at 25 °C; fumaric acid: 0.6 gr/100 mL, at 25 °C. These two isomers have the same number of hydrophilic groups, but their solubility differs by a factor of more than 100. Similar differences are observed in various isomers of dicarboxylic benzene.

Two more problems in protein chemistry, which were known for a long time as the most difficult problems in biochemistry, are the protein folding problem and the self-assembly of proteins. Both of these phenomena were known to occur in aqueous solutions. For some time, it was believed that hydrophobic interactions were responsible for these processes (Kauzmann [13], Tanford [14], Dill [15], Delvin [16], Voet and Voet [17], and Whitford [18]). However, in the late 1990s, following the study of the solvation of proteins and examination of the various solute-induced interactions between various hydrophilic groups, it was concluded that the hydrophilic, not hydrophobic, interactions are probably the most important factor in determining the Gibbs energy changes for these processes. For more details on all these topics, the reader is referred to reference [4].

## Figures and Tables

**Figure 1:**
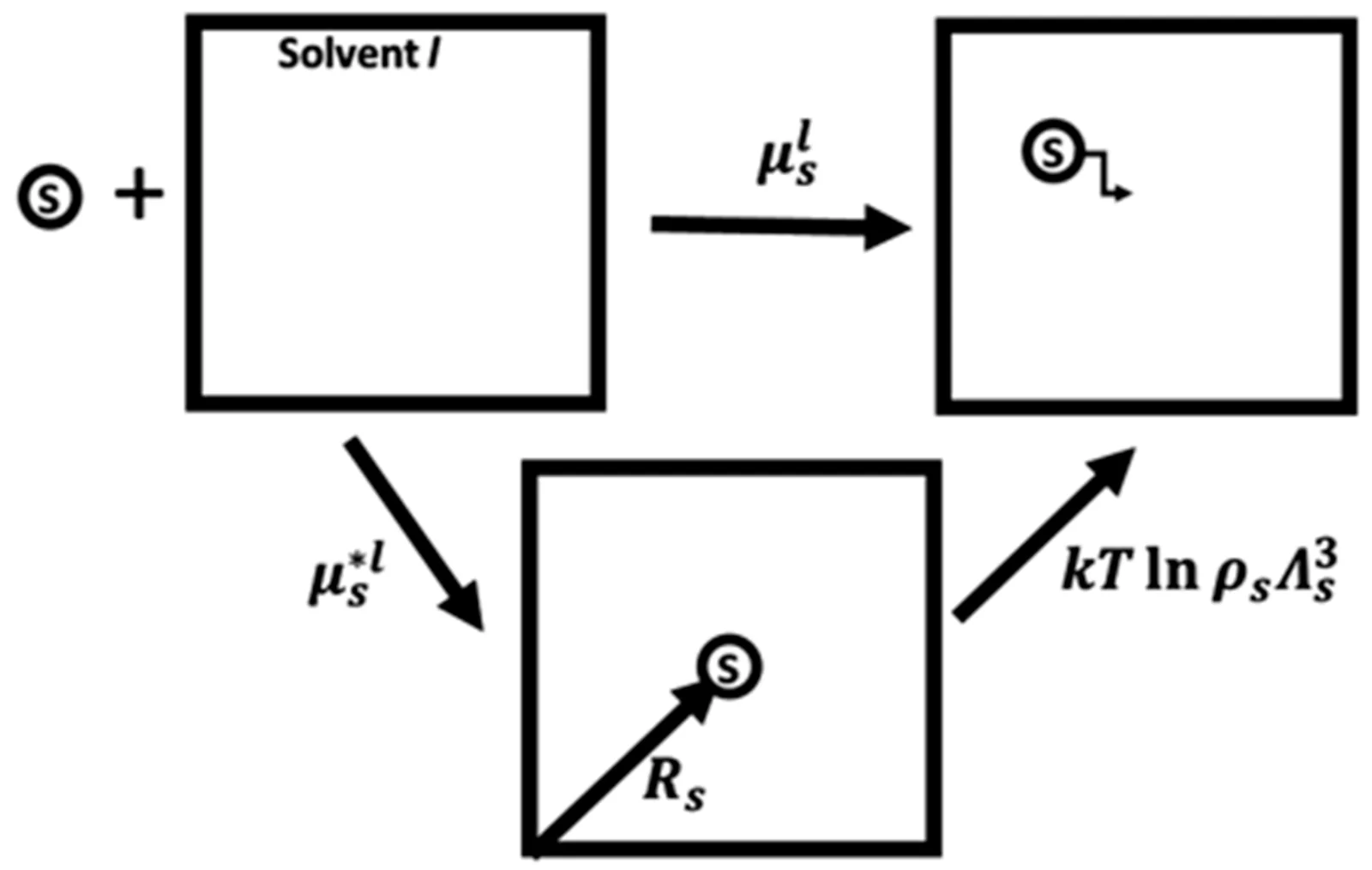
The chemical potential of a molecule s is split into two terms: First, the work associated with placing the molecule at a fixed position ($R_{s})$ in the solvent $\left(\mu_{s}^{*l}\right)$, and second, the change in Gibbs or Helmholtz energy for releasing the molecule from the fixed position: ($kT\ln\rho_{s}\Lambda_{s}^{3})$.

**Figure 2:**
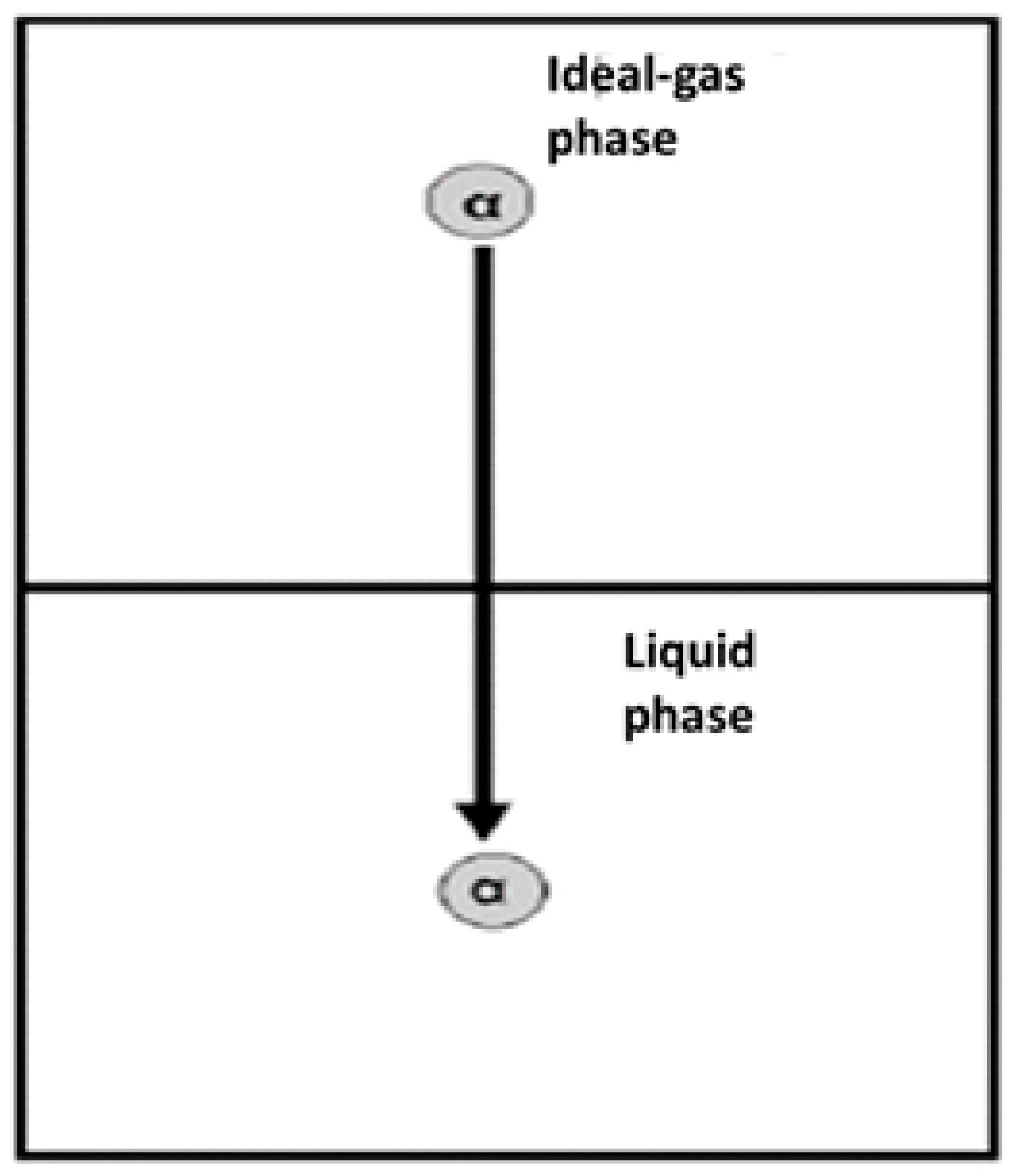
Definition of the process of solvation: A molecule of species α is transferred from a fixed position in an ideal-gas phase, into a fixed position in the liquid phase.

**Figure 3:**
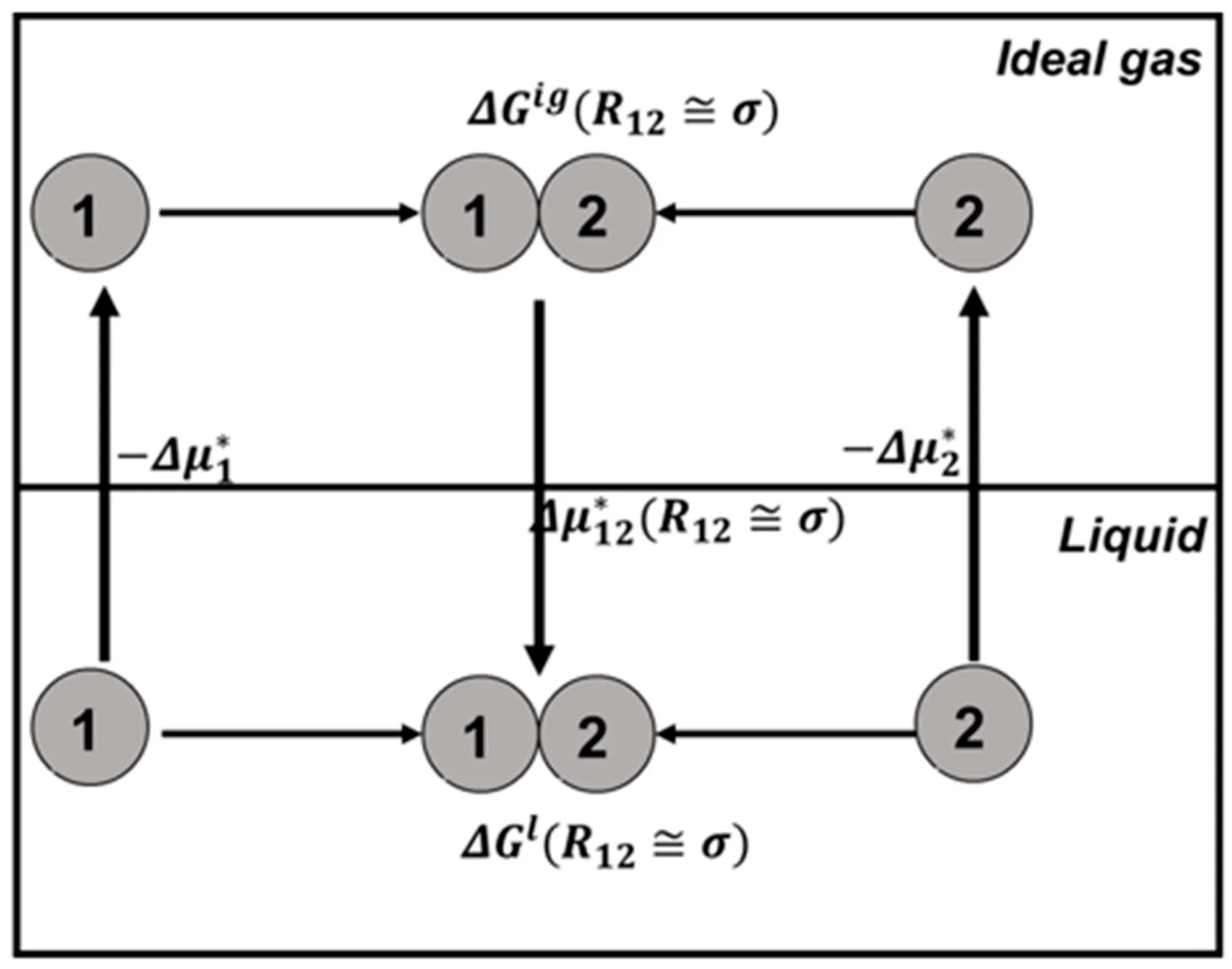
The process of bringing the two solute particles 1 and 2 from infinite separation ($R_{12}=\infty$) to some finite distance $R_{12}\;≅\;\sigma$. The same process is carried out in an ideal-gas and in a liquid phase.

## Data Availability

No new data were created or analyzed in this study. Data sharing is not applicable to this article.

## References

[1] Gurney R.W. (1953). Ionic Processes in Solution.

[2] Ben-Naim A. (2006). Molecular Theory of Solutions.

[3] Ben-Naim A. (1987). Solvation Thermodynamics.

[4] Ben-Naim A., Sordo J.A. (2026). Modern Solvation Thermodynamics.

[5] Ben-Naim A. (2009). Molecular Theory of Water and Aqueous Solutions: Part I: Understanding Water.

[6] Ben-Naim A. (1974). Water and Aqueous Solutions, Introduction to a Molecular Theory.

[7] Eley D.D. (1939). On the Solubility of Gases. Part I.—The Inert Gases in Water. Trans. Faraday Soc..

[8] Eley D.D. (1944). The Structure of Water in Aqueous Solutions. Trans. Faraday Soc..

[9] Frank H.S. (1945). Free Volume and Entropy in Condensed Systems I. General Principles. Fluctuation Entropy and Free Volume in Some Monatomic Crystals. J. Chem. Phys..

[10] Frank H.S., Evans M.W. (1945). Free Volume and Entropy in Condensed Systems III. Entropy in Binary Liquid Mixtures; Partial Molal Entropy in Dilute Solutions; Structure and Thermodynamics in Aqueous Electrolytes. J. Chem. Phys..

[11] Hughes W.L., Saroff H.A., Carney A.L. (1949). Preparation and Properties of Serum and Plasma Proteins. XXII. A Crystallizable Guanidinated Derivative of Human Serum Albumin. J. Am. Chem. Soc..

[12] Wang H., Ben-Naim A. (1997). Solvation and Solubility of Globular Proteins. J. Phys. Chem. B.

[13] Kauzmann W. (1959). Some factors in the interpretation of protein denaturation. Adv. Protein Chem..

[14] Tanford C. (1973). The Hydrophobic Effect.

[15] Dill K. (1990). Dominant Forces in Protein Folding. Biochemistry.

[16] Delvin M.T. (2006). Textbook of Biochemistry with Clinical Correlations.

[17] Voet D.J., Voet J.G., Pratt C.W. (2008). Principles of Biochemistry.

[18] Whitford D. (2005). Proteins: Structure and Function.

